# Effect of shared decision making on mode of delivery and decisional conflict and regret in pregnant women with previous cesarean section: a randomized clinical trial

**DOI:** 10.1186/s12884-021-03615-w

**Published:** 2021-02-17

**Authors:** Fatemeh Hadizadeh-Talasaz, Faezeh Ghoreyshi, Fatemeh Mohammadzadeh, Roghaieh Rahmani

**Affiliations:** 1 Department of Midwifery, Faculty of Medicine, Social Development & Health Promotion Research Center, Gonabad University of Medical Sciences, Gonabad, Iran; 2 Student Research Committee, Faculty of Medicine, Gonabad University of Medical Sciences, Gonabad, Iran; 3 Department of Epidemiology & Biostatistics, School of Health, Social Development and Health Promotion Research Center, Gonabad University of Medical Sciences, Gonabad, Iran

**Keywords:** Shared decision making, Vaginal birth after cesarean, Repeated cesarean section, Conflict, Regret

## Abstract

**Background:**

The promotion of vaginal birth after cesarean section (VBAC) is the best method for the reduction of repeated cesarean sections. Nonetheless, the decisional conflict which often results from inadequate patient involvement in decision making, may lead to delayed decision making and regret about the choices that were made. The present study aimed to determine the effect of shared decision making on the mode of delivery and decisional conflict and regret in pregnant women with previous cesarean section.

**Methods:**

This randomized clinical trial was conducted on 78 pregnant women with a previous cesarean section referring to community health centers in Torbat-e Jam, Iran, in 2019. They were randomly assigned to two groups of intervention and control. During weeks 24-30 of pregnancy, the Decisional Conflict Scale (DCS) was completed by pregnant mothers. Apart from the routine care, the experimental group received a counseling session which was held based on the three-talk model of shared decision making. This session was moderated by a midwife; moreover, a complementary counseling session was administered by a gynecologist. During weeks 35–37 of pregnancy, DCS was completed, and the Decision Regret Scale (DRS) was filled out for both groups at the 8th weeks postpartum and they were asked about the mode of delivery. Data were analyzed in SPSS software (version 19) using the Mann-Whitney, Chi-squared and Fisher’s exact tests. *p*-value less than 0.05 was considered statistically significant.

**Results:**

After the intervention, the decisional conflict score was significantly lower in the shared decision making (SDM) group, compared to that in the control group (14.90 ± 9.65 vs. 25.41 ± 13.38; *P* < 0.001). Moreover, in the SDM group, the rate of vaginal birth was significantly higher than that in the control group (P < 0.001). Two month after the delivery, the mean score of decision regret was lower in the SDM group, in comparison to that in the control group (15.67 ± 23.37 vs. 27. 30± 26.75; *P* = 0.007).

**Conclusions:**

Based on the results of the study, shared counseling can be effective in the reduction of decisional conflict and regret, as well as rate enhancement of VBAC. Therefore, it can be concluded that this counseling method can be used in prenatal care to reduce the rate of repeated cesarean section.

**Trial registration:**

IRCT20190506043499N1; Name of the registry: Iranian Registry of Clinical Trials; Registered 10.

August 2019. URL of registry: https://en.irct.ir/trial/39538. Date of enrolment of the first participant to the trial: August 2019.

## Background

The increasing rate of cesarean section has evoked a global health concern. As evidenced by the recent data, approximately 50% of cesarean sections are performed selectively, and the majority of these operations are due to repeated cesarean section [1]. The promotion of vaginal birth after cesarean (VBAC) for eligible women and rate enhancement of successful VBACs are the best methods for the reduction of repeated cesarean sections [2]. Although ample evidence demonstrated that VBAC is a safe mode of delivery for most women, and 3 out of 4 women may be successful, VBAC rates are declining [1].

The rate of trial of labor after cesarean (TOLAC) and VBAC vary widely across the globe [3]. VBAC rates were reported as 29-36% in Ireland, Italy, and Germany, 45–55% in Finland, Sweden, and the Netherlands, 14% in Australia [4], and 13.3% in the United States [5]. The Healthy People 2020 goals include an increase in VBAC to 18.3% [6, 7]. Decision making regarding the mode of delivery (e.g., vaginal birth or cesarean section) is one of the most challenging decisions for pregnant mothers and the medical team. Due to the complex nature of decision making process, appropriate and effective tools are needed to improve and facilitate decision making. These tools can involve pregnant women in decision making, increase information, and reduce concerns [8]. One of these tools is shared decision making (SDM) which is a dynamic and interactive process in which the patient and health care providers share information. In so doing, they can have shared treatment decision making based on the best available evidence, as well as patient values and preferences [9–12].

Patients’ participation and awareness of their preferences improve the quality of care and treatment outcomes [13]. The results of a systematic review (2007) demonstrated that patients with greater participation in treatment decisions were more informed of their options and had realistic expectations about what might happen. Moreover, the majority of them selected the option which was most valuable to them [14]. Previous data suggested that the degree of decisional conflict experienced by patients may be affected by the degree of shared decision making in patient counseling. The patients who experience more SDM will have less decisional conflict [15].

Decisional conflict in patients often results from insufficient patient involvement in decision making, which may lead to delayed decision making and decision regret [16]. Shared decision making using decision aids is one of the techniques for the reduction of decision conflict in primary care. Decision regret can also potentially be modified through shared decision making [17]. Hong et al. (2016) suggested that more involvement in decision making process will reduce decisional conflict and regret [11].

Shared decision making can help and facilitate decision making; nonetheless, no study was retrieved from databases on the impact of shared decision making on decisional conflict and regret in pregnant women with a previous cesarean section in Iran. According to the aforementioned issues, the present study aimed to determine the effect of shared decision making on mode of delivery and decisional conflict and regret in women with a previous cesarean section.

## Methods

This randomized clinical trial with two parallel groups (intervention and control groups) was performed on pregnant women in Torbat-e Jam, Iran, from August 2019 to March 2020. The minimum sample size was calculated at 78 subjects; however, a total of 86 cases (*n* = 43 in each group) were entered into the study considering 10% sample attrition. This calculation was performed based on a similar study [18], the mean difference formula, as well as considering the effect size d = 0.75, type I error of 0.05, and the test power of 80% using G*Power software (version 3.1.9.2). The sample size was calculated for both conflict and regret variables, and finally, a larger sample size was considered.

The inclusion criteria were as follows: 1) women with singleton pregnancies with live fetuses, 2) absence of fetal anomalies, 3) no medical problems (including diabetes, hypertension, as well as heart, liver, and kidney disease), 4) history of only one previous cesarean section, 5) previous transverse cesarean scar, 6) absence of contraindication to vaginal delivery in current pregnancy (large fetus, stable placenta previa, multiple births, polyhydramnios and oligohydramnios, eclampsia and preeclampsia, rupture of membrane, unreliable fetal condition, non-cephalic presentation), 7) gestational age of 20-30 weeks, 8) interval between the previous delivery and the first day of the last menstrual period of the current pregnancy more than 6 months, 9) waiting to give birth in 37 weeks or more, 10) absence of mental illness, and 11) non-reception of written advice other than the usual counseling of the center. On the other and, the exclusion criteria entailed: 1) unwillingness to continue cooperation, 2) occurrence of medical or obstetric contraindications to vaginal delivery, 3) delivery before the end of the study period, 4) absence in any of the two counseling sessions, 5) participation in other counseling sessions other than the usual counseling of the center, and 6) incomplete questionnaires in any of the follow-up stages.

Data collection tools included: demographic and obstetric profile questionnaire, Decisional Conflict Scale (DCS) developed by O’Connor, Decision Regret Scale (DRS), and a question about the mode of delivery .

DCS is a 16-item self-report questionnaire with five subscales, including informed, values clarity, social support, uncertainty, and effective decision [19]. Participants respond to each item using a 5-point Likert scale ranging from 0 (strongly agree) to 4 (strongly disagree). In each of the subdomains or subscales, firstly, the scores of the items are summed up, divided by the number of items, and multiplied by 25. The total score ranges from 0 to 100. A score below 25 means the implementation of the decision, and a score of ≥37.5 signifies decision delay or feeling unsure about implementing their decisions [20]. This questionnaire is standard, and its validity and reliability have been confirmed in several studies. For instance, in a study conducted by Moudi, the reliability of this scale has been confirmed rendering a Cronbach’s alpha coefficient of 0.92 and a correlation coefficient of 0.99 [18]. In the present study, the reliability of the questionnaire was confirmed by the internal consistency method rendering a Cronbach’s alpha coefficient of 0.81.

DRS is a five-item scale that measures regret after treatment decisions at a given point in time [21]. The participants respond to each item using a 5-point Likert scale ranging from 1 (strongly agree) to 5 (strongly disagree) [22]. Items 2 and 4 are reversely scored. The score of each item is subtracted from 1 and then multiplied by 25. To obtain the final score, the items are summed and averaged. The final score falls within the range of 0 to100. A score of 0 means no regrets, while a score of 100 signifies high regret [22, 23]. The validity and reliability of the abovementioned scale have been investigated in numerous studies. For example, in a study conducted by Moudi, reliability was confirmed with Cronbach’s alpha coefficient of 0.94 and a correlation coefficient of 0.99 [18]. The reliability of the scale in the present study was confirmed by the internal consistency method rendering a Cronbach’s alpha of 0.87.

To select the samples, firstly, four centers were randomly selected from the community health centers of Torbat-e Jam. Considering that pregnant mothers in each of the centers may communicate with each other and be informed of the shared decision making and intervention, mothers in two centers were randomly assigned to the control group and their counterparts in the other two centers, who were similar to the subjects in the first center in terms of location and socioeconomic status, were allocated to the case group. Thereafter, using the pregnant mothers’ registration system, eligible women with a previous cesarean section were listed and invited via phone call to participate in the study. A total of 86 eligible pregnant women who were willing to participate were included in the study. They were randomly assigned to intervention and control groups (*n* = 43 in each group).

Thereafter, mothers in the experimental group were provided with necessary information about the counseling session at the next visit (week 24-30). At weeks 24-30 of gestation, demographic and fertility characteristics form as well as DCS were completed by pregnant mothers. In addition to routine care in both groups, the experimental group received a 90-min counseling session based on the three talk model of shared decision making [12] with the presence of the researcher, pregnant mother, spouse, and other people desired by the pregnant mother or her husband.

In the first stage (choice talk), the patient received the necessary information about the types of options (VBAC and repeated cesarean section). In the second stage (option talk), patients’ information concerning the main options and participants’ narratives were examined. In the third stage (decision talk), the pros, cons, risks, and family costs associated with each option were discussed, women with successful VBAC were interviewed, the couple’s values/ preferences and concerns were talked over, and patients were supported to make a decision. If needed, more information was provided to mothers and their companions, and the final decision was left to mothers and their companions (Table 1). In the counseling session, apart from being provided with needed information, pregnant mothers were acquainted with the delivery department of Torbat-e Jam. After the first consultation session, other sessions were held if necessary. The counseling was conducted by a graduate student who was a midwife working in the delivery department and had received the necessary training on counseling. At the end of the session, each mother received a pamphlet on the advantages and disadvantages of VBAC. Moreover, the researcher’s phone number was handed to the intervention group to contact the researcher in case they had any questions. Moreover, the researcher referred the pregnant mothers in the intervention group and their companions to one of the gynecologists in order to provide them with the necessary information to complete the consultation (second session) and answer their possible questions. At weeks 35–37 of gestation, DCS was completed in both intervention and control groups. In addition, DRS was completed for mothers in both groups 8 weeks after delivery in community health centers, and they were asked about the mode of delivery. The current study covered 24-30 weeks of gestation to two months after childbirth.

**Table 1:** Contents of two sessions of shared decision making (SDM) counseling

Stages of three-talk model of SDM	Essential elements	Consultant responsibilities		
Stage 1. Choice talk	Problem presentation	According to the history of cesarean section, evaluation the mode of delivery in the current pregnancy		
	Available options	It is time to think about what mode of delivery you will choose in the future: vaginal delivery or cesarean section		
Stage 2. Option talk	Evaluation of mothers and companions’ information regarding the mode of delivery in women with previous cesarean section	Before making any decision, ask them to explain their information about the mode of delivery in women with previous cesarean section (Try to deeply understand their main narrations)	- Checking information -Examining a clear understanding of information (Is their information correct or misunderstood?)	- Providing more information - Answering questions -Providing evidence if necessary
Stage 3. Decision talk	1- Discussing the pros/cons /risks/and family expenses	Using participants’ explanations and narratives to understand and extract points related to: physical, psychological, financial, and social impact of vaginal delivery/cesarean section on both family and newborn	- Checking information -Examining a clear understanding of information (Is their information correct or misunderstood?)	-Providing more information -Answering questions -Providing evidence if necessary -Interviews with people with successful vaginal birth after cesarean section
	2- Modification of values/preferences of mother and companions	Using the participant’s explanations and narratives to extract and clarify what is most important to them.	1. Listing the most important values, concerns, and worries of mother and companions 2. Helping them to have accurate and realistic preferences	-The consultant helps them to predict what they prefer to happen in the future. Moreover, how do they feel the short-term and long-term consequences
	3. Asking about mothers’ decisions and companions	They are asked: Are you ready to decide? Or do you need more time?	Sometimes they explicitly need more time. The counselor examines the reasons and asks if they have any further questions. Are there any other things that people have heard or read about on the Internet and should be discussed?	1.Providing an opportunity to talk to a gynecologist about new advances in delivery methods 2- Presenting the opinion of the American College of Obstetricians and Gynecologists about the method of delivery after cesarean section 3.Providing evidence if necessary
	4. Discussing patient’s abilities and companions	They are asked: Are you sure you made up your mind?	Sometimes they delay the decision. The counselor should look into their reasons and whether they have more questions.	1.Providing more information 2. Answering questions 3.Providing evidence if necessary
	5. Follow-up	Follow-up 8 weeks after delivery to review the decision		

Data were analyzed in SPSS (version 19) using Chi-square, Mann-Whitney, Fisher’s exact tests. *p*-value less than 0.05 was considered statistically significant.

CONSORT guidelines were adhered on reporting this clinical trial.

## Results

**Fig. 1:**
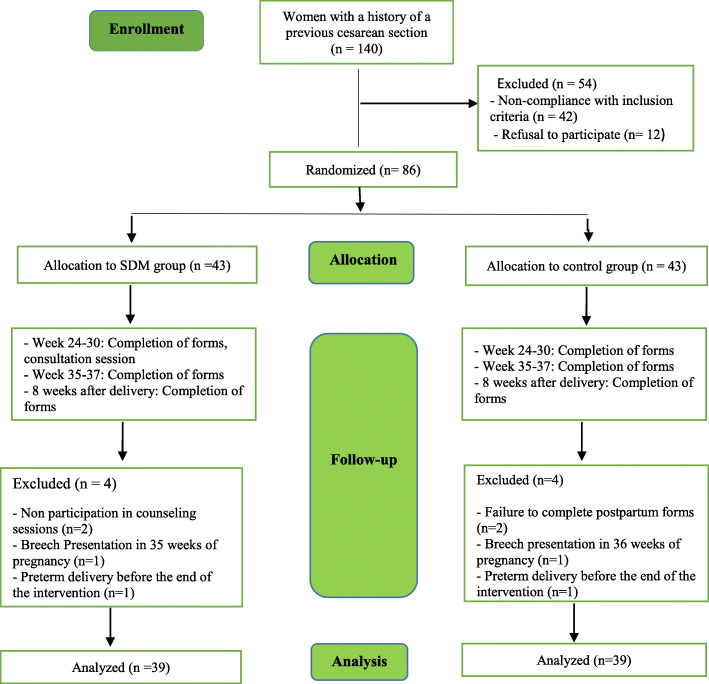
Flowchart of study participants

During the study, four participants were excluded from the intervention group due to different reasons, including not attending counseling sessions (*n* = 2), delivery before the end of the intervention (preterm delivery) (*n* = 1), fetal indication for cesarean section (breech presentation at 35 weeks of pregnancy) (*n* = 1). Moreover, four cases were ruled out from the control group due to different reasons, including incomplete forms after delivery (n = 2), fetal indication for cesarean section (breech presentation at 36 weeks of gestation) (n = 1), delivery before the end of the intervention (preterm delivery) (n = 1). Finally, 78 people entered the study (Fig. 1).

The results of data analysis on the personal and midwifery characteristics of the participants demonstrated that the mean age of mothers in the SDM group and control group were 29.58 ± 4.60 years and 30.48 ± 3.79 years, respectively. The mean years of schooling of mothers in the SDM group and the control group were reported as 10. 30±2.05 and 11.38 ± 3.86 years, respectively. The mean of the inter-pregnancy interval in the SDM group and control group were 5.11 ± 2.02 and 5.74 ± 2.71 years. The mean number of pregnancies in the SDM and control group were reported as 3.02 ± 1.22 and 2.66 ± 0.88. The result of the Mann-Whitney test showed that there was no statistically significant difference between the two groups in terms of the abovementioned variables (*P* > 0.05). Other characteristics of the participants are presented in Table 2. The two groups were homogeneous regarding of confounding factors such as the type of previous cesarean section, the indication for previous cesarean section, wanted pregnancy and received pregnancy care.

**Table 2:** Comparison of personal and midwifery characteristics of participants in SDM and control groups

Characteristic		SDM group	Control group	Total	P-value*
		N (%)	N (%)	N (%)	
Occupation	Housewife	29 (74.3)	26 (66.7)	55 (70.5)	0.32^a^
	Employee	9 (23.1)	13 (33.3)	22 (28.2)	
	Laborer	1 (2.6)	0 (0)	1 (1.3)	
	Total	39 (100)	39 (100)	78 (100)	
Previous cesarean section	Emergency	34 (87.2)	33 (84.6)	77 (30.8)	0.33^a^
	Elective	5 (12.8)	6 (15.4)	11 (69.2)	
	Total	39 (100)	39 (100)	78 (100)	
Indication of previous cesarean section	Abnormal fetal presentation	20 (58.8)	18 (54.6)	38 (56.7)	0.08^a^
	Lack of labor progress	7 (20.6)	8 (24.2)	15 (22.4)	
	Abnormal fetal heart rate	4 (11.8)	6 (18.1)	10 (14.9)	
	Excretion of meconium in amniotic fluid	1 (2.9)	1 (3.1)	2 (3.0)	
	Placenta Previa	2 (5.9)	0 (0.0)	2 (3.0)	
	Total	34 (100)	33 (100)	67 (100)	
Wanted pregnancy	Yes	30 (76.9)	31 (79.5)	61 (78.2)	1.00^b^
	No	9 (23.1)	8 (20.5)	17 (21.8)	
	Total	39 (100)	39 (100)	78 (100)	
Regular pregnancy care	Yes	38 (97.4)	38 (97.4)	76 (97.4)	1.00^b^
	No	1 (2.6)	1 (2.6)	2 (2.6)	
	Total	39 (100)	39 (100)	78 (100)	

*Significance level: P < 0.05

^a^Chi-squared test

^b^Fisher’s exact test

Before the intervention, the mean total scores of decisional conflict in the SDM and control groups were obtained at 30.51 ± 11.26 and 28.41 ± 15.01, respectively, and no significant difference was observed between the groups (*P* = 0.29). Nonetheless, after the intervention, the mean total score of decisional conflict in the SDM group (14.90 ± 9.65) was significantly lower, as compared to that in the control group (25.41 ± 13.38). The scores of subscales (informed, values clarity, social support, uncertainty, and effective decision) were also significantly lower in the SDM group, in comparison to those obtained in the control group (Table 3).

**Table 3:** Comparison of decisional conflict and regret scores in SDM and control groups

Decisional conflict score		SDM group	Control group	P-value**
		Mean (SD*)	Mean (SD)	
**Subscales**				
Informed	Before intervention	29.04 (8.51)	30.98 (17.93)	0.54
	After intervention	9.61 (11.23)	20.93 (15. 27)	0.001
Values clarity	Before intervention	32.47 (16.75)	32.69 (15.92)	0.77
	After intervention	19.65 (13.31)	27.54 (17.10)	0.03
Social support	Before intervention	27.98 (14.99)	22.56 (16.07)	0.17
	After intervention	8.33 (10.46)	16.66 (12.82)	0.002
Uncertainty	Before intervention	31.67 (17.67)	26.54 (17.76)	0.18
	After intervention	9.40 (10.32)	19.22 (16.57)	0.009
Effective decision	Before intervention	30.82 (19.26)	25.80 (17.74)	0.28
	After intervention	9.77 (10.80)	16.45 (13.09)	0.03
Total score	Before intervention	30.51 (11.26)	28.41 (15.01)	0.29
	After intervention	14.90 (9.65)	25.41 (13.38)	< 0.001
**Decisional regret score**		15.67 (23.37)	27. 30 (26.75)	0.007

*Standard Deviation

**Significance level: P < 0.05

** Mann–Whitney U-test

Out of 24 mothers in the intervention group who were hospitalized for the trial of labor after cesarean, five mothers underwent emergency cesarean section due to lack of progress in delivery (*n* = 2), abnormal fetal heart rate (n = 2) and meconium excretion in amniotic fluid (*n* = 1). In the control group, out of 11 mothers who were hospitalized for the trial of labor, three mothers underwent emergency cesarean section due to lack of progress in delivery (n = 2) and abnormal fetal heart rate (n = 1). Finally, the results indicated that 19 (48.7%) participants in the SDM group and 8(20.5%) cases in the control group gave birth vaginally. Chi-squared test denoted that there was a significant difference between the mode of delivery in the two groups (*P* < 0.001; Table 4).

**Table 4:** Comparison of the trial of labor and mode of delivery in SDM and control groups

Variable			SDM group	Control group	Total	P-value^a^
			N (%)	N (%)	N (%)	
Trial of labor after cesarean (yes)			24 (61.5)	11 (28.2)	35 (44.8)	< 0.001
Mode of delivery	VBAC^c^		19 (48.7)	8 (20.5)	27 (34.6)	< 0.001
	RCS^d^	Emergency	5 (12.8)	3 (7.7)	8 (10.3)	
		Elective	15 (38.5)	28 (71.8)	43 (55.1)	

^a^Significance level: P < 0.05

^a^Chi-squared test

^c^Vaginal birth after cesarean

^d^Repeated cesarean section

The mean scores of decision regret two month after delivery were 15.76 ± 23.37 and 27. 30±26.75 in the SDM and the control group, respectively. Furthermore, the results of the Mann-Whitney test revealed that there was a statistically significant difference between the two groups in terms of decision regret score (*P* = 0.007; Table 3).

Regarding intra and postpartum complications, the results of the research showed that in the intervention group, there was one case of uterine dehiscence, two cases of postpartum hemorrhage, three cases of low neonatal Apgar score, two cases of infant hospitalization in the intensive care unit and one case of postpartum fever. In the control group, there was one case of postpartum hemorrhage, two cases of low neonatal Apgar score, two cases of infant hospitalization in the intensive care unit and two cases of postpartum fever. No maternal or fetal death was reported.

## Discussion

As evidenced by the results of the present study, shared decision making, along with the use of pamphlets, reduced the total score of decisional conflict below the threshold of 25. In other words, mothers who attended SDM counseling sessions had lower decisional conflict about the mode of delivery. On the other hand, they had a greater awareness of the benefits and risks of delivery modes, social support, and appropriate decision making. Furthermore, individual values were taken into account to a greater extent in decision making.

This finding assumes great significance since decisional conflict causes people to change their minds, delay their decision, and make decisions with undesirable outcomes [17, 18] and regret [24]. Based on the results of a study carried out by Shorten et al. (2005), the intervention group who received decision aid showed a reduction in decisional conflict, compared to the control group [25].

Montgomery et al. (2007) performed a study to assess the effects of two computer-based decision aids on decisional conflict and mode of delivery in women with a previous cesarean section. The results of the mentioned study illustrated that there was less decisional conflict in the intervention group, compared to that in the control group, and the difference between them was significant [26]. In a study conducted by Eden et al. (2014), the use of decision tools (decision aids or brochures) helped women reduce conflict over birth decisions. These products provide the needed information about the benefits and risks of modes of delivery; nonetheless, they differ in the format and level of risk detail [27]. The results of a study performed by Moudi et al. (2018) indicated that the total score of decisional conflict, as well as the scores of subscales (informed, values clarity, social support, uncertainty, and effective decision), were significantly lower in the intervention group, compared to those obtained in the control group [18]. The findings of the present study are in line with those obtained in the aforementioned studies. Therefore, it can be stated that shared decision making increases the knowledge of pregnant women and their husbands, answer their questions, and involve them in the decision making process. Therefore, it is effective in the reduction of decisional conflict, enhancement of decision making quality, and ease of making this choice among the available options. In a study conducted by Kuppermann et al. (2020), patient-centered decision support tools had no effect on decisional conflict [28]. The results of the referred study are inconsistent with the findings reported in the present study. This difference can be ascribed to the gestational age when the intervention was performed. In the mentioned study, the intervention was performed before the 25th week of pregnancy, while it was conducted after the 25th week in the present study. Moreover, in the stated study, most of the participants had higher education and there was relatively high rate of trial of labor in both randomization groups. Furthermore, tablet-based decision aids were used in the abovementioned study, whereas in the present study, face-to-face consultation was conducted. Various formats, such as pamphlets, interactive media, video, or audio-tapes are not a good substitute for physician consultation. Moreover, face-to-face consultation offers a wide range of possibilities for discussion, information exchange, and effective supportive interventions. This explanation highlights the effectiveness of our counseling in the reduction of decisional conflict. Furthermore, face-to-face counseling based on shared decision making opens up the possibilities of information provision, communication, and discussion even for people with low literacy. McCaffrey et al. (2007) in their systematic review of patient decision making tools reported that patients and physicians who used decision aids made better decisions. Patients with more involvement in treatment decisions were more informed about their options and had more realistic expectations about what might happen; moreover, the majority of them selected the option that was most valuable to them and better suited to their conditions [14].

Based on the results of the present study, shared counseling increased the rate of VBAC so that there was a significant difference between the mode of delivery in the two groups. Hamilton et al. (2016) noted that a good medical decision is achieved with a good decision making process [29]. The findings of a study carried out by Wise et al. (2019) indicated that women who were initially uncertain about their preferred mode of delivery showed a greater reduction in decisional conflict score after receiving additional decision aid and were more likely to plan for VBAC [30]. However, inconsistent with the findings of the present research, the results of the study conducted by Kuppermann et al. (2020) on the effect of a patient-centered decision support tool revealed that the rate of vaginal delivery was not significantly different between the intervention and control groups [28]. This discrepancy in results can be attributed to the gestational age when the intervention was performed. In the abovementioned study, the intervention was performed before the 25th week of pregnancy, while it was conducted after the 25th week in the present study.

In the present study, the level of decision regret in the first month after delivery was lower in mothers who participated in SDM counseling sessions. This finding is significant since regret weakens the intention to repeat the same choice [18].

Although birth usually ends in a positive outcome, many women experience negative emotions [31], especially when their delivery is not as expected [32]. Negative experiences before, during, or following decisions about treatment measures can lead to regret about the choices that were made [22]. The results of a study conducted by Becerra-Perez et al. (2016) showed that most patients who received primary care indicated mild regrets about the decision and experienced more regrets in the event of decisional conflict [17]. The findings of the stated study are in compliance with the present study, the intervention group who received shared counseling along with the pamphlet reported less decisional conflict and regret. In a study conducted by Konheim-Kalkstein et al. (2019) entitled “Regrets from women with an unplanned cesarean delivery”, the results illustrated that 73% of women expressed a feeling of regret after childbirth, and only emotional support was accompanied by less regret [33]. In agreement with the present research, in the study by Moudi et al. (2018), at 1-month post-abortion, the regret score was low which can be ascribed to the pre-abortion counseling session, as well as shared decision making [18]. Ghiasvandian et al. (2013) carried out a study on the effect of decision aids on decision regret in patients with breast cancer after 8 weeks of treatment. The results denoted that there was no statistically significant difference between the two groups in terms of regret. It can be attributed to the short follow-up duration after treatment initiation which did not allow for tracking the positive or negative effects of this choice (the type of treatment) on quality of life and health consequences of patients during this short period [34]. The results of the mentioned study are not consistent with those obtained in the present study.

The notable strengths of the present study include the use of shared counseling for mothers with previous cesarean sections who were a sensitive group in need of counseling process, the creation of great opportunities for mothers with successful VBAC to attend counseling sessions, and the presence of pregnant women’s relatives in counseling sessions. On the other hand, the current study had several limitations. The First limitation was the unpredictability of healthy pregnant women to continue participating in the study due to prenatal problems and exclusion from the study. Secondly, due to the nature of the study and the fact that the intervention and data collection was performed by one of the authors, it was not possible to blind the participants and the data collector. Thirdly, it was not possible to use a larger sample size due to time constraints. Fourthly, although the content of the decision aid was carefully designed so as not to advocate from a particular delivery method and the researchers also paid attention to this point during the consultation, but counselor bias during the directed counseling was one of the limitations of the study.

## Conclusion

The overall results of the present study demonstrated that SDM counseling sessions increase awareness, value clarity, as well as decision support. In so doing, it can be of great help in the reduction of decisional conflict and regret, as well as the rate enhancement of VBAC. In other words, shared decision making helps counselors engage mothers and spouses in a thoughtful discussion so that they can make a realistic and defensible decision with the least likelihood of regret. Therefore, this counseling method can be used in prenatal care to reduce the rate of repeated cesarean section.

## Data Availability

Additional information is not available to maintain confidentiality.

## Abbreviations

VBAC: Vaginal birth after cesarean
TOLAC: Trial of labor after cesarean
RCS: Repeated cesarean section
SDM: Shared decision making
DCS: Decisional conflict scale
DRS: Decision regret scale
